# Oil Palm Waste-Based Precursors as a Renewable and Economical Carbon Sources for the Preparation of Reduced Graphene Oxide from Graphene Oxide

**DOI:** 10.3390/nano7070182

**Published:** 2017-07-13

**Authors:** Salisu Nasir, Mohd Zobir Hussein, Nor Azah Yusof, Zulkarnain Zainal

**Affiliations:** 1 Materials Synthesis and Characterisation Laboratory (MSCL), Institute of Advanced Technology (ITMA), Universiti Putra Malaysia, Serdang, Selangor 43400, Malaysia; salisunasirbbr@gmail.com (S.N.); azahy@upm.edu.my (N.A.Y.); zulkar@upm.edu.my (Z.Z.); 2 Department of Chemistry, Faculty of Science, Federal University Dutse, 7156 Dutse, Jigawa State, Nigeria

**Keywords:** oil palm wastes, carbonization, graphitic carbon, graphene oxide, reduced graphene oxide, Raman spectroscopy

## Abstract

Herein, a new approach was proposed to produce reduced graphene oxide (rGO) from graphene oxide (GO) using various oil palm wastes: oil palm leaves (OPL), palm kernel shells (PKS) and empty fruit bunches (EFB). The effect of heating temperature on the formation of graphitic carbon and the yield was examined prior to the GO and rGO synthesis. Carbonization of the starting materials was conducted in a furnace under nitrogen gas for 3 h at temperatures ranging from 400 to 900 °C and a constant heating rate of 10 °C/min. The GO was further synthesized from the as-carbonized materials using the ‘improved synthesis of graphene oxide’ method. Subsequently, the GO was reduced by low-temperature annealing reduction at 300 °C in a furnace under nitrogen gas for 1 h. The I_G_/I_D_ ratio calculated from the Raman study increases with the increasing of the degree of the graphitization in the order of rGO from oil palm leaves (rGOOPL) < rGO palm kernel shells (rGOPKS) < rGO commercial graphite (rGOCG) < rGO empty fruit bunches (rGOEFB) with the I_G_/I_D_ values of 1.06, 1.14, 1.16 and 1.20, respectively. The surface area and pore volume analyses of the as-prepared materials were performed using the Brunauer Emmett Teller-Nitrogen (BET-N_2_) adsorption-desorption isotherms method. The lower BET surface area of 8 and 15 m^2^ g^−1^ observed for rGOCG and rGOOPL, respectively could be due to partial restacking of GO layers and locally-blocked pores. Relatively, this lower BET surface area is inconsequential when compared to rGOPKS and rGOEFB, which have a surface area of 114 and 117 m^2^ g^−1^, respectively.

## 1. Introduction

Green technology is generally considered as a strong stimulant towards economic growth and a means for mitigating environmental degradation, especially in developing countries. Malaysia is one of the world’s largest palm oil producers with about 950,000 ha of land [1] and oil palm industries; the up- and down-streams are a story of success. In the past forty years, palm oil has witnessed a remarkable and sustained growth in the global market. It is estimated that annual production of palm oil in Malaysia may reach about 15.4 million tonnes between 2016 and 2020 [1,2]. In essence, much lignocellulosic biomass is generated every day from the oil palm industries. This includes oil palm trunks (OPT), oil palm fronds (OPF), empty fruit bunches (EFB) and palm pressed fibers (PPF), palm kernel shells (PKS), palm oil mill effluent palm (POME), etc. It is alarming that wastes generated from oil palm activities are posing major disposal problems [2,3]. Consequently, there is a need for technological, cost-effective, energy balance and environmental considerations in a balanced proportion in order to resolve utilization of oil palm wastes.

For this reason and coupled with our quest and curiosity to explore new knowledge, we felt it was essential to devise an experimental means of enhancing the utilization and production of profitable materials from those ‘green wastes’ generated by oil palm industries that can match the ‘wastes to wealth’ mantra, which is focused towards ‘zero-waste industry’.

Interestingly, this is occurring also at a time when the world is moving towards green technology. The good prospects of natural precursors as a source of carbon are that they are not only renewable, but also inexpensive and have the potential to be the green alternatives for industrial-scale production of carbon nanomaterials, such as graphene, graphene oxide (GO), reduced graphene oxide (rGO), graphene quantum dots (GQDs), carbon nanotubes (CNT), activated carbon (AC), etc. [4,5].

In general, carbon nano-structured materials, particularly graphene and other graphene-related and derived materials, are characterized by an extraordinary permutation of exceptional properties developed after a wide range of applications predominantly in areas such as electronics, photonics, energy [6] and environmental sensing [7]. The recent advancement of material science has strongly been linked to a broad range of useful properties pertaining to the thermal resistance, electrical conductance and extraordinary strength of these carbon nanomaterials [8]. This has paved the way for their use as excellent materials for different types of industries [9].

Many companies have developed a high level of concern in the processing, modification and customization of these materials and thus have posed a strong need for a method that can be used to characterize these materials. Hence, Raman spectroscopy is one technique that has proven to be appropriate for many of the characterization needs with these materials [10]. For this reason, it was used in this work in concert with X-ray diffraction (XRD) and many other state-of-the-art equipment for the characterization of the as-prepared GO and rGO materials. Various techniques such as high-resolution transmission electron microscopy (HRTEM), scanning electron microscopy (SEM) and others have also been used in the past to study the structural changes of carbon nanomaterials (graphite and nitrogen-doped carbon nanoparticles) during ball milling [11]. Concurrently, the X-ray diffraction system has commonly been used to characterize the structures of hexagonal graphitic and turbostratic carbon [12]. Both the latter and the former techniques usually impose very little constraint on the substrate size during analysis and are generally nondestructive.

In spite of its short history, the popularity of graphene and its derivatives such as GO and rGO is tremendously spreading every day. This owed to its intense strength and raging electrical, optical and mechanical properties [13,14]. The wonders and prospects of these nanomaterial have been extensively reported and widely publicized as the next big thing in everything from high-frequency transistors and photo-detectors, to flexible electronics and biosensors [14,15].

It is noteworthy that other semiconductor materials have shown similar structural properties to graphene. Some of them are single crystalline semiconductor films derived from approximately 1 mm-thick, single crystalline wafer templates [16]. Unfortunately, they were very costly and not reusable. The advent of graphene and its derivatives is now a miracle, as it can be produced in the laboratory from commonly-available and inexpensive bio-agricultural by-products (as we did in this project), hence replacing those expensive materials for different type of applications [4,5,17,18]. Correspondingly, the application and uses for graphene and other graphene-derived materials have been tested and reported for a number of electronics [9]. The ability of electrically-charged particles to move frequently through a medium in response to an electronic field makes graphene a promising material for electronics applications [9,19]. It has been forecasted that graphene and other graphene-based materials could be used as transparent electrodes for touch screen devices, rollable e-paper and foldable light emitting diodes (LEDs) in the very near future [13]. Concomitantly, many other applications and uses are underway for large-area graphene in high-frequency transistors, logic transistors/thin-film transistors, etc.

In this present study, graphene oxide and reduced graphene oxide were successfully synthesized. Captivatingly, graphene oxide is generally considered as the most widely-used substance to produce graphene-like material on a large scale [20]. The chemical oxidation of graphite flakes to give a non-stoichiometric compound formerly known as graphitic oxide or graphitic acid was first reported by an Oxford chemist (Benjamin C. Brodie) about 150 years ago by treating graphite with a mixture of potassium chlorate and fuming nitric acid [21]. In 1958, Hummers and Offeman came up with a procedure (popularly known as Hummer’s method) [22] that was considered to be more proficient, fast and safer by using a mixture of H_2_SO_4_, NaNO_3_ and KMnO_4_ to oxidize graphite. Since then, much interest was directed to oxidized graphite due to its incredible mechanical, thermal and electrical properties [23,24,25]. It is believed that the presence of reactive oxygen functional groups in the GO molecule is the driver for all of these excellent properties. To further enhance the new properties of GO, it can be dispersed in water and other organic solvents.

Despite all of the good prospects of the GO as the most resourceful and processable graphene precursor, however, there are several challenges in the industrial-scale production of this material, especially in the influence of carbon or graphite source. This drawback has hindered rapid commercialization of GO as compared to chemical vapor deposition (CVD)-graphene due to its complexity. For this reason, GO is mostly processed in combination with other materials, rather than as a finished product for a market with an explicit demand, and its application is also narrowed to where defect-free graphene is not essential [26]. Depending on the application, graphene oxide is usually subjected to additional transformation like bio-conjugation for biomedical-related applications, reduction for appropriate conductivity-related application, etc. For this reason and coupled with the specific demands of different industrial sectors, the production of graphene oxide is expected to be tailored in a way that it will suit all of these kinds of demands.

More so, in spite of all of the promising future of GO for different types of applications due to its incredible processability and dispersibility, it has been reported that the existing chemical oxidation procedures to produce GO are limited by some drawbacks, especially in the formation of permanent hole defects on the GO sheet and residual metal ions contaminations [27]. This has made some researchers explore other greener approaches, such as electrochemical methods that do not require extensive purification steps during the synthesis process. However, in our literature survey, we realized that most of the reported work on the electrochemical approach used single preformed bulk graphite as the electrode, which also confines their reproducibility, scalability, as well as the degree of oxidation. Nonetheless, some groups of researchers had recently come up with a mechanically-assisted electrochemical method to fabricate graphene oxide directly from graphite flakes [28]. They claimed that the electrochemically-derived graphene oxide had a good degree of oxidation with also less physical defects than the chemically-derived graphene oxide.

In light of the aforementioned properties, the material is now used in many types of applications. The most prominent ones are, but not limited to, sensors [29], polymer composites, field effect transistors, energy related materials and biomedical applications [24,30,31,32,33]. Moreover, due to the disrupted sp^2^ bonding networks in graphene oxide, it is also used as an insulating material [34,35], although it can easily be modified to a conductive material by introducing it into reducing agents [28,29]. Therefore, removing oxygen groups from GO platelets (i.e., reduction reaction) is also one of the most vital and interesting reactions of graphene oxide. The material generated when GO is reduce has a remarkable feature resembling pristine graphene. The deoxygenation or reduction of GO to produce rGO or to a graphene-like material is mostly carried out using different methods [20,31,32]. The most notable ones are chemical and thermal reduction methods [36].

In a chemically-mediated reduction, a variety of chemicals has been used to remove the oxygen groups from the GO [37]. Hydrazine hydrate was the first reported and most widely-used chemical to accomplish the task [38]. Other strong reducing agents such as lithium aluminum hydride (LiAlH_4_), sodium borohydrate (NaBH_4_) [39], etc. were also used. However, these strong reducing agents react belligerently with the water molecule, which is widely used as the dispersing agent for the GO. Comparatively, hydrazine monohydrate does not have such strong reactivity with the water, hence making it a good option for removing oxygen groups in the aqueous dispersion of GO. Nevertheless, concerns about the harmful effects regarding the chemical toxicity of hydrazine have also been raised, and therefore, many other ‘green’ preferences have been designed and reported in order to replace hydrazine hydrate for the chemical reduction of graphene oxide [31,38,39,40,41].

The other category or means of reducing the GO is through heat treatment. For example, there are numerous thermally-mediated methods to reduce GO in which the oxide functionalities on the GO are stripped from the surface by directly heating the GO in the furnace under certain conditions and atmosphere [42]. This method is capable of producing a thermodynamically-stable carbon dioxide (CO_2_) species. The stacked structure is exfoliated by extrusion of the CO_2_, which is produced when the GO is heated between 200 and 1050 °C. It has been observed that the high temperature gas created intense pressure within the stacked layers. For example, Gonzalez et al. observed structural damage to the GO platelets as a result of the CO_2_ extrusion during the thermal exfoliation process. In addition, a significant mass of the GO is lost to that effect, and topological defects and vacancies were formed in the rGO structure [38].

The most commonly-used material for GO synthesis is high pure graphite flake [43]. Few researches had attempted to use other natural and industrial carbonaceous wastes to synthesize graphene oxide using a variety of methods. In our literature survey, we found that Akhavan et al. had used these materials to produce GO and rGO [44]. In their procedure, the modified Hummers method was adopted to synthesize the GO and rGO. They obtained a graphitic material that served as the precursor to the former and the latter synthesis by wrapping-up the raw materials in aluminum foil and scorching in a reduced oxygen condition for five days, milled into fine powders, further annealed at (450 °C) overnight and diffused in an aqueous solution of FeCl_3_·6H_2_O and HCl. Their method, though sounding promising, was perceived as tedious and time consuming, and the precursors used are not one hundred percent economical.

Another group of researchers, Adolfsson et al., reported to have transformed cellulose materials generated from waste paper into carbon nanospheres via the hydrothermal method [45]. The degradation of the rich cellulose content in the waste paper was conducted in a microwave device at 160 °C with sulfuric acid serving as the catalyst. To further obtain the GO quantum dots, the carbon nanospheres, which served as the precursor, were sonicated and heated in 70% HNO_3_ solution [45]. In relation to these reports, few works can be found in the literature that mainly correlated to the use of biomass wastes to synthesize high quality and large area graphene; an example can be found in [46].

Nevertheless, we were compelled to carry out this research because the mining of high purity graphite flakes, which comprise the foremost raw material utilized to synthesize graphene oxide and reduced graphene oxide, are polluting and very costly as compared to the industrial graphite. Secondly, the cost of graphene oxide in the market is approximately $200 per gram [47], although some manufacturers in Asia (e.g., Six Element, China) are selling their industrial-grade GO for as little as $400 per kilogram. For this reason and coupled with the vast availability of oil palm waste in Malaysia, we felt it was necessary to derive a means of harnessing these materials (that are generally considered unimportant) into prosperity. To the best of our knowledge, no work was reported, particularly using oil palm leaves, palm kernel shells and empty fruit bunch precursors, aimed at GO and rGO preparations as we did in this project.

## 2. Materials and Methods

### 2.1. Sample Collection and Preparation

The biomass materials used as precursors in this research work were natural or bio-agricultural waste generated from palm oil plantation industries in (Selangor, Malaysia), one of the most abundant biomass resources in the country. The palm kernel shell (PKS) and empty fruit bunch (EFB) were collected from Sri Dewin Oil Palm Mill Sdn. Bhd. (Dengkil, Selangor, Malaysia), while oil palm leaves (OPL) were collected from Taman Pertanian Universiti, Ladang 8, Universiti Putra Malaysia, (Serdang, Selangor, Malaysia).

All of the samples were thoroughly washed several times with deionize water, dried in an oven at 70 °C and crushed into a powder.

### 2.2. Carbonization

**Figure 1:**
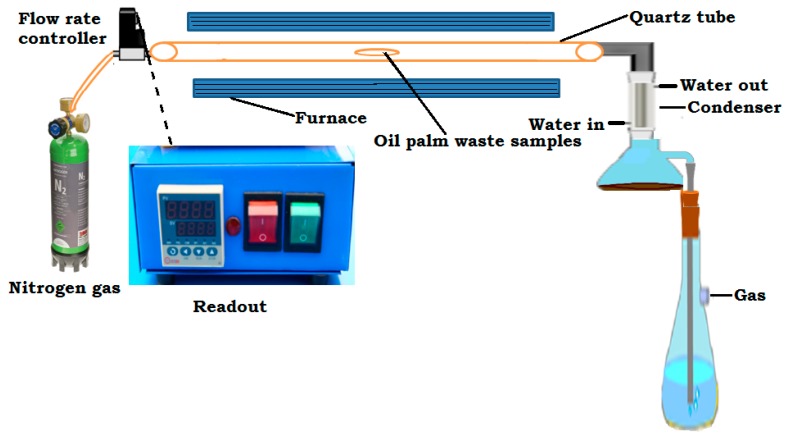
A schematic illustration of the experimental setup for samples carbonization using oil palm waste precursor.

The carbonization or analytical pyrolysis of the starting material was conducted in a furnace under an inert atmosphere of nitrogen gas (as illustrated in Figure 1) to obtain a carbon material that served as the precursor for rGO synthesis. About 6.0 g of the powdered sample were weighed and transferred into an alumina boat, placed in a quartz tube and into the midpoint of the furnace chamber. Nitrogen gas was allowed to flow through the system throughout the carbonization period. The precursors OPL, PKS and EFB were heated at a constant heating rate of 10 °C/min, to the temperatures of 400, 500, 600, 700, 800 and 900 °C and held for 3 h for optimization. After keeping the final temperature for 3 h, the heating process was stopped. Concurrently, the nitrogen gas flow was maintained until the chamber temperature dropped to 100 °C. After the sample temperature reduced to room temperature, the sample was weighed again to determine the yield percentage. Finally, the as-carbonized materials, i.e., carbon material, were made into powder using a mortar and pestle and transferred into sample bottles (ready for further analysis) prior to rGO synthesis. The as-synthesized materials were analyzed using Raman Spectroscopy, X-ray diffraction and other characterization methods. 

### 2.3. Materials Synthesis

The graphene oxide synthesis was carried out following the ‘improved graphene oxide synthesis’ as reported by Marcano et al. [43]. One key advantage with this synthesis method is that the reaction is not exothermic and does not produce toxic gases, thus making it suitable for our objective. After the synthesis, the as-produced materials were washed twice repeatedly in 200 mL of deionized water, 200 mL of 37% HCl and 200 mL of ethanol, each step followed by centrifugation. The materials after this repeated washing process were further coagulated with 200 mL of diethyl ether, and the resulting solutions were centrifuged and the supernatant (excess ether) decanted away. The residue was carefully removed from the centrifuging tubes and dried overnight at room temperature, obtaining about 5.00 g of the GO products.

The as-prepared GOs were subsequently reduced through thermal treatment using low-temperature annealing reduction of the graphene oxide [42,48]. The thermal annealing reduction was conducted at 300 °C in a furnace and operated in an inert atmosphere of nitrogen gas flow.

All of the reagents and chemicals utilized in this work were of analytical grade and were used directly without further purification.

### 2.4. Characterization

The Raman spectroscopic analysis of all of the prepared samples was conducted using a ‘Wissenschaftliche Instrumente und Technologie’ (WITec) Raman spectrometer, Alpha 300R (WITec GmbH, Ulm, Germany). The Raman frequency was acquired with a laser excitation wavelength of 532 nm and an integration time of 5.03645 (s). The intensity ratio between the D-band at around 1365 cm^−1^ and the G-band (~1585 cm^−1^) of the Raman spectra was used to measure the graphitic character of the as-prepared materials. X-ray diffraction analyses of the as-produced materials were conducted using a powder X-ray diffraction (XRD-6000 diffractometer, Shimadzu, Tokyo, Japan) at room temperature. The scanning range of all the samples was 5 to 40° (2θ), with a scanning rate of 4° min^−1^. The external surface morphology and microstructure of all of the rGOs were examined using a field emission scanning electron microscope (FESEM) using an FEI Nova NanoSEM 230, (FEI, Hillsboro, OR, USA). This machine has two operating vacuum modes (high vacuum and low vacuum) to deal with different types of samples and can achieve a magnification of over 500,000×.

The surface functional groups of the GOs and different rGOs were investigated using Fourier transform infrared spectroscopy (FTIR) technique using Thermo Nicolet, Nicolet 6700 model, (Thermo Scientific, Waltham, MA, USA), at a resolution of 4 cm^−1^ over the range of 400 to 4000 cm^−1^. The samples were mixed with potassium bromide (KBr) pellets to drop off the concentration of the sample and acquire a better spectrum [49]. Thus, pellets were prepared from the mixture of 1 mg of the sample and 200 mg of KBr. This mixture was compacted in a manual hydraulic press. The FTIR spectra and the absorbance were then recorded in the range of 400 to 4000 cm^−1^, and the analysis was carried out at room temperature. Moreover, the surface area and pore volume of the samples were characterized by BET and N_2_ adsorption-desorption isotherm analyses using BELSorp Mini II (NIKKISO, Osaka, Japan). Finally, the Thermogravimetric analysis (TGA) and its derivative (DTG) were conducted using a TGA/DSC 1HT model, (METTLER TOLEDO, Shah Alam, Selangor, Malaysia) to measure the changes in properties of the as-prepared materials as they are heated, and was carried out within a temperature range between 25 and 1000 °C.

## 3. Results and Discussion

### 3.1. Raman Spectroscopy and the Informational Evidences as Revealed by the I_D_/I_G_ Ratios

**Figure 2:**
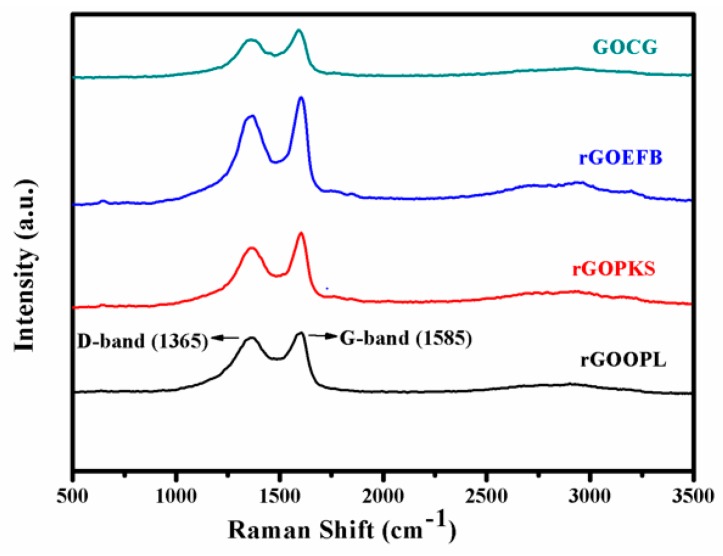
Raman spectra of the GO and rGO samples prepared from four different precursors; oil palm leaf (OPL), palm kernel shell (PKS), empty fruit bunch (EFB) and commercial graphite (CG).

Raman spectroscopy is one of the most widely-used analytical techniques to characterize the bonding structure of carbon-based materials. The technique usually imposes very little constraint on the substrate size and is generally nondestructive [50]. As one of the characterization technique used in this study, all of the resulting materials (rGOOPL, rGOPKS, rGOEFB and GOCG) were characterized by this method. The lattice distortions were confirmed from the Raman spectra, as shown in Figure 2, which illustrate D- and G-bands at 1365 cm^−1^ and 1585 cm^−1^, respectively. The G-band of the as-prepared GO shifted towards a higher wavenumber, indicating the oxidation of graphite-like material, which results in the formation of sp^3^ carbon atoms. However, the D-band is relatively broadened owing to the size reduction of the in-plane sp^2^ domains at the time of oxidation.

Apparently, Tuinstra and Koenig were the first to come up with the idea of using the intensity of the D-band to that of the G-band (I_D_/I_G_) to assess the extent of the graphite cluster size [51]. The correlation that exists between the I_D_/I_G_ ratios measured from the Raman spectrum and the graphitic in-plane microcrystallite size, La, obtained from X-ray diffraction was very much taken into consideration in their studies. Concomitantly, a linear correlation between I_D_/I_G_ and 1/La was observed and reported following a sequence of measurements on microcrystalline graphite samples with varying microcrystalline sizes [42,44]. Several other works have effectively used the I_D_/I_G_ ratio to evaluate La while investigating properties, such as the degree of disorder in amorphous carbon, nanographite crystal size and the domain for glassy carbon films [52]. However, it has been proven difficult to make direct correlations when the I_D_/I_G_ value outsized the maximum value observed in the microcrystalline graphite studies. Consequently, the linear relationship between the I_D_/I_G_ and the inverse of the microcrystallite size 1/La does not occur when the value is greater than 1.1 [53,54]. Comparatively, as the number of graphite microcrystallites with fewer defects (instead of the increase in La) increases, the I_D_/I_G_ ratio also increases. This also explains the increase in the intensity of D-band for most amorphous films after annealing at a higher temperature [38,42,44,46]. It has been reported that a higher heating temperature increases the short-range order, but not La [37,45,46]. Similarly, it has been inferred from the Raman spectra of graphene and other amorphous carbon that as a disorder in graphene increases, the I_D_/I_G_ undergoes two different behaviors. One of these behaviors is seen at a regime of ‘diminutive’ defect density where I_D_/I_G_ will increase as a higher defect density generates ancillary elastic scattering. On the other hand, at a regime of high defect density, the I_D_/I_G_ will start to reduce as an increasing defect density result in a more amorphous carbon structure, hence reducing all Raman bands [42,44,47].

**Figure 3:**
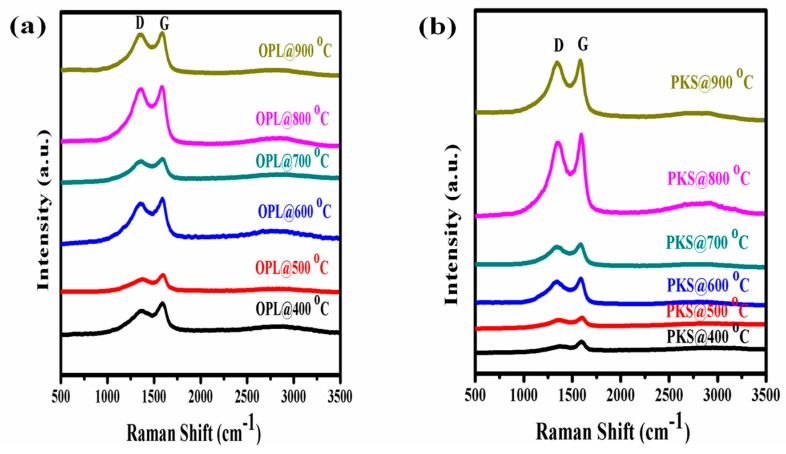
Raman spectra of the carbonized oil palm leaf (OPL) and palm kernel shell (PKS) at various carbonization temperatures during optimization. The D- and G-bands at around 1365 cm^−1^ and 1585 cm^−1^, respectively, provide evidence for the graphitic nature of the sample.

In this work, different annealing temperatures were used to prepare the graphite-like material prior to the GO and rGO synthesis. Interestingly, for all of the heating temperatures, the analyzable Raman spectrum was obtained. The spectra of oil palm leaves (OPL) at various carbonization temperatures, 400, 500, 600, 700, 800 and 900 °C, were virtually similar in comparison to that of palm kernel shell (PKS), but always lower in terms of I_D_/I_G_. Moreover, the Raman bands become more prominent with increasing the annealing temperature in the majority of the samples (Figure 3).

However, it is worth noting that we took into account the Raman spectral pattern of other related carbon-based materials from previous literature for comparison. Mostly, the G-band disperses in more disordered carbon, and the dispersion is proportional to the degree of disorder, but it does not disperse in graphite, glassy carbon or nano-crystalline graphite [55]. The Full width at half maximum (FWHM) of the peak decreases with increasing excitation energy because of the gap variation of the carbon films. However, it should be noted that even for the same sample, the Raman peak position and width may slightly vary with the laser excitation wavelength. In general, the I_D_/I_G_ ratio or its reciprocal (I_G_/I_D_) acquired with laser excitation in the visible region is usually used to substantiate the degree of graphitization of carbon-based materials [44,46]. Therefore, we initially considered these ratios as a criterion for determining the optimum temperature condition for graphitic carbon formation. In the same way, the I_G_/I_D_ values (Table 1) were also used to specify the graphitization level of the as-synthesized materials.

**Figure 4:**
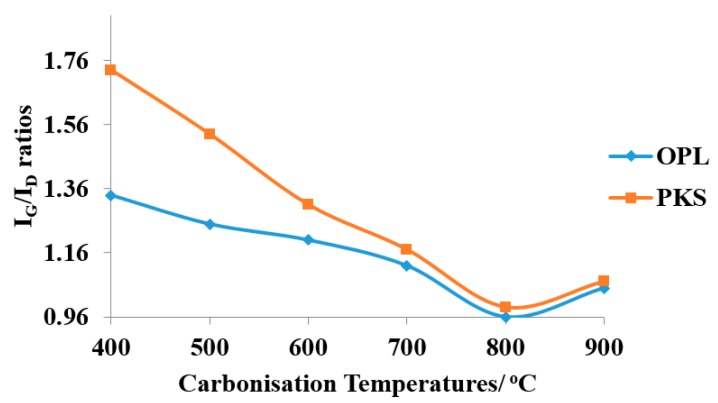
Plot of I_G_/I_D_ ratios against carbonization temperatures of the as-produced graphite-like material synthesized from oil palm leaf (OPL) and palm kernel shell (PKS) precursors.

**Figure 5:**
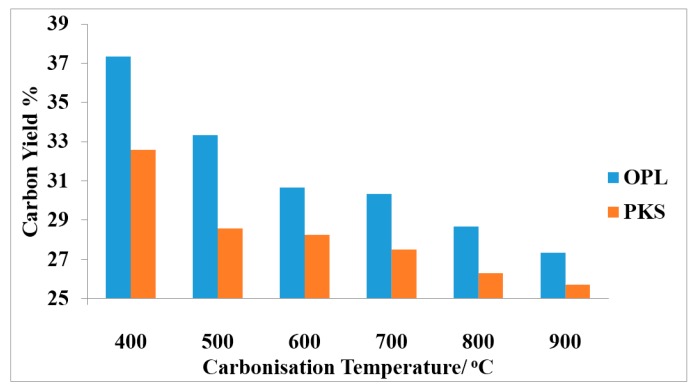
Carbon yield % prepared from precursors; OPL and PKS against carbonization temperatures showing that the yield tends to decrease as the carbonization temperature increased.

It is important to note that the initial investigation of the effect of heating temperature on the formation of graphitic carbon and the yield was limited to oil palm leaves and palm kernel shell raw samples only prior to their consequential GO and rGO synthesis (Figure 3, Figure 4 and Figure 5). The limitation was based on the fact that both OPL and PKS have comparable densities to that of empty fruit fiber and commercially-obtained graphite (CG), respectively. These had significantly reduced the superfluous waste of energy that is needed to carbonize the materials and also the bulk experimental work associated with them. Nevertheless, all of the samples, oil palm leaves (OPL), palm kernel shells (PKS), empty fruit fiber (EFB) and commercially-acquired graphite (CG), were fully utilized in all of the remaining analyses as elucidated elsewhere in this article.

The plot of I_G_/I_D_ ratios against carbonization temperatures generated from the two different samples is shown in Figure 4. The formation of graphitic phase shows a different slope, which indicates that the rate of the graphitic formation from the precursors was higher in PKS than OPL, and they are temperature dependent almost linearly up to 800 °C. Generally, palm kernel shell (PKS) is relatively denser than oil palm leaf (OPL). This presumably contributed to the easier formation of the graphitic phase of the PKS than the OPL, as indicated by the plot. However, we observed no crucial difference between the samples (precursors) after heating above 800 °C, and the ratios were found to optimally converge at 900 °C, evidence of the graphitic nature formation. Thus, we deduce that relatively high temperature treatment is needed to remove nearly all of the non-graphitic phase in the resulting samples.

### 3.2. X-ray Diffraction Analysis

**Figure 6:**
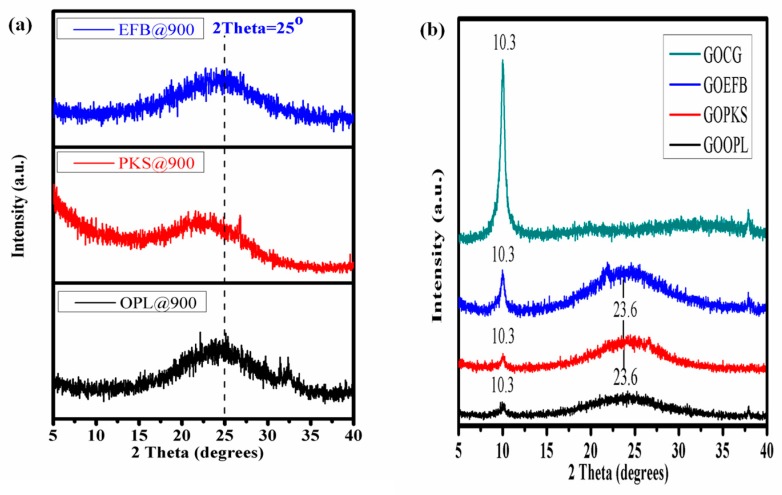
X-ray diffraction patterns of (**a**) graphite-like material produced at 900 °C from the various precursors; (**b**) GO samples prepared from the as-produced graphite-like materials generated from the various precursors.

**Figure 7:**
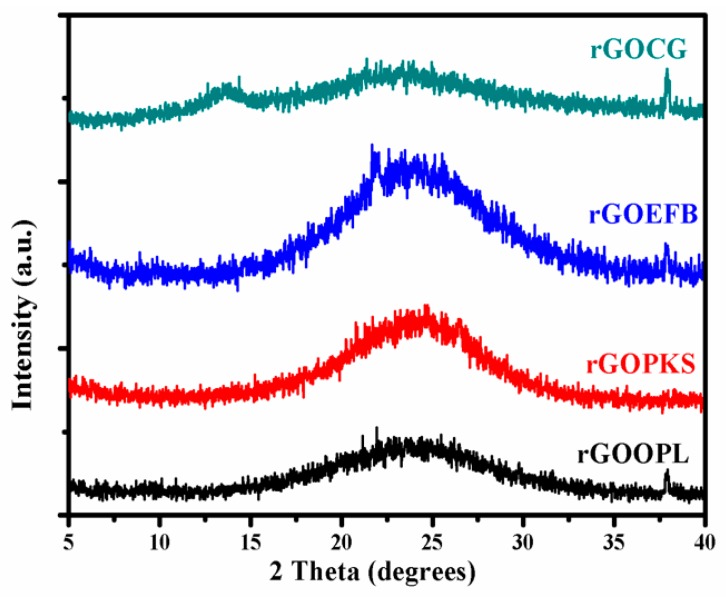
XRD patterns of rGO samples obtained after the low-temperature annealing reduction of the graphene oxides prepared using various precursors.

The X-ray diffraction pattern (Figure 6 and Figure 7) shows the overall oxidation and reduction of the samples. The technique was employed to examine the crystallographic phase of the GOs and rGOs (rGOOPL, rGOPKS, rGOEFB and GOCG). Initially, the as-prepared graphite-like material exhibits a basal reflection (002) peak at 2θ = 25° as illustrated in Figure 6a. On oxidation of the graphite, the 002 reflection peak shift to the lower angle (2θ = 10.3°).

Therefore, the strong and weak diffraction peaks observed at 2θ = 10.3° (Figure 6b) signify GO formation due to chemical oxidation of the as-prepared graphite-like material and commercially-obtained graphite, respectively. This chemical oxidation introduces oxygen functionalities to the plane of the graphitic carbon and, therefore, the degree of oxidation is proportional to the interlayer spacing of the materials. The increase in d-spacing is undoubtedly owing to the intercalation of H_2_O molecules and the emergence of oxygen-containing functional groups between the layers of the graphite sheets. However, the peak observed at 2θ = 10.3° completely disappeared following the low-temperature annealing reduction of the graphene oxide (Figure 7). The peak at 2θ = 23 to 25° emerged owing to the formation of rGO. This spectacular shift to elevated 2θ angles (23.6° and 25.3°), which is indicative of lattice d-spacing, was observed for all the non-commercial rGO (i.e., rGOOPL, rGOPKS, rGOEFB) samples. Conversely, a decrease of the d-spacing could possibly suggest a better ordering of the two-dimensional structure of the materials [56]. On the other hand, an increase in lattice spacing in rGO indicates the presence of oxygen functional groups [57]. Consequently, the broad peak observed in these samples (at 2θ angles = 23.6°) could possibly be attributed to few layers of rGO sheets in the particles, which connote the presence of the multilayer domains of the rGO sheets.

### 3.3. FTIR Spectroscopy Analysis

**Figure 8:**
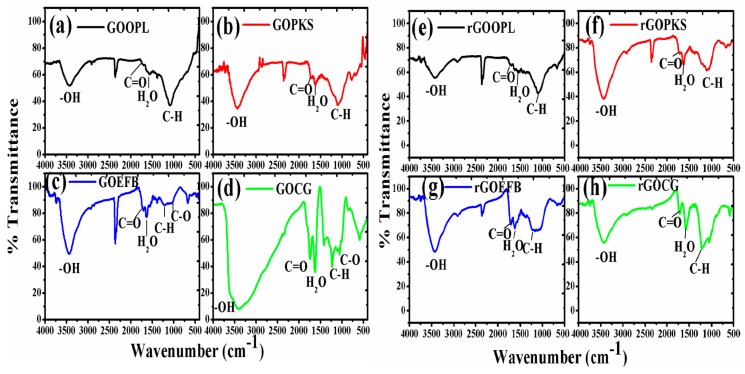
Comparison of the FTIR spectra of the GOs (**a**–**d**) and rGOs (**e**–**h**) samples.

The FTIR spectrum of the GO and rGO samples is presented in Figure 8. However, the FTIR spectrum of rGOs was relatively different from that of GO after the low-temperature annealing reduction. The intensities of absorption bands corresponding to oxygen-containing groups were considerably decreased, indicating a nearly complete reduction. In both cases, the following functional groups were all recognized in the samples after the conversion of raw precursors to GO and to rGOs. The band at 3442 cm^−1^, which exist due the O–H stretching vibration in rGOCG, was weaker than O–H vibration in the FTIR spectra of the GOCG, this may perhaps be due the waning of hydroxyl groups and removal of volatile molecules of the reduced samples. The band at 1628 cm^−1^ was attributed to C=O stretching vibration. Similarly, a band at around 1640 cm^−1^ in the GOs and rGOs spectra (Figure 8) was attributed to water molecule bending modes [58]. Other strong bands were observed at 1560 cm^−1^, which is attributed to the intense stretching of conjugated C–C in the aromatic ring or oxygen-aromatic bonding in the aromatic ether. Furthermore, the peak at 1383 cm^−1^ was assigned to C–H bending vibrations. Moreover, a band at 1039 to 1218 cm^−1^ was also observed, which include the reduction of ester, carboxylic acids and alcohol.

### 3.4. FESEM Analysis

**Figure 9:**
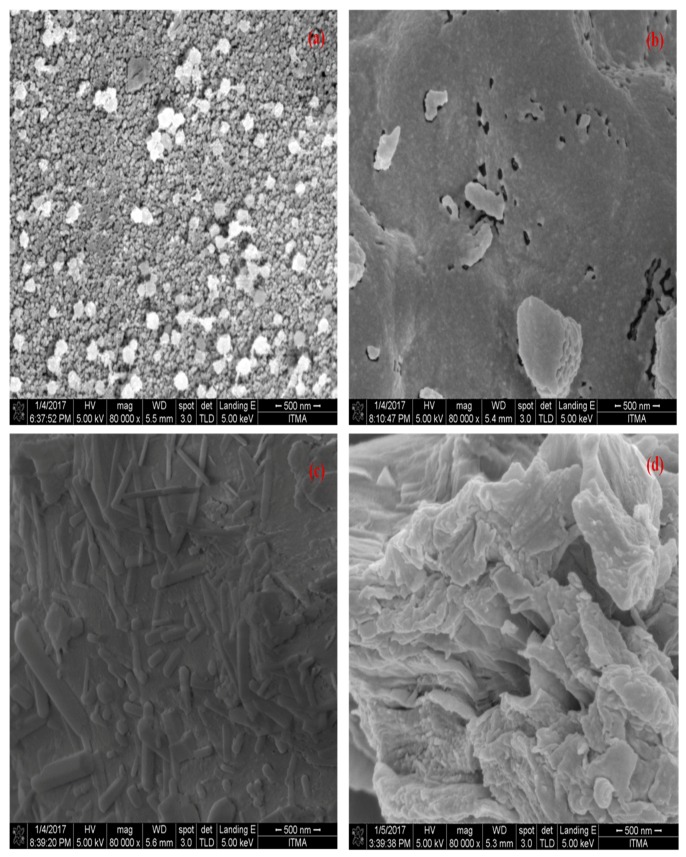
FESEM micrographs of the as-prepared rGOs and GO samples: (**a**) rGOOPL, (**b**) rGOPKS, (**c**) rGOEFB and (**d**) GOCG displaying dispersed multi-layered rGO nanoballs and GO flake-like structures.

Figure 9 shows the FESEM micrographs for the rGOs and the GO samples prepared from different precursors. Micrographs were recorded with 80,000× magnification. Pores of heterogeneous shapes and sizes were observed due to the different nature of the starting materials. The pores could be primarily caused by quick gas evolution under the high vapor pressure generated while centrifuging and washing with the concentrated HCl (37 wt%) after the GO synthesis process [59]. It can be seen in Figure 9a–c that rGOOPL, rGOPKS and rGOEFB produced small pores and nanoball like-structure distributed heterogeneously. These small pores with distinct sizes developed within the bigger pores. On the other hand, in Figure 9d, porosities were reduced and left only flake-like carbon.

### 3.5. Thermogravimetric Analysis

**Figure 10:**
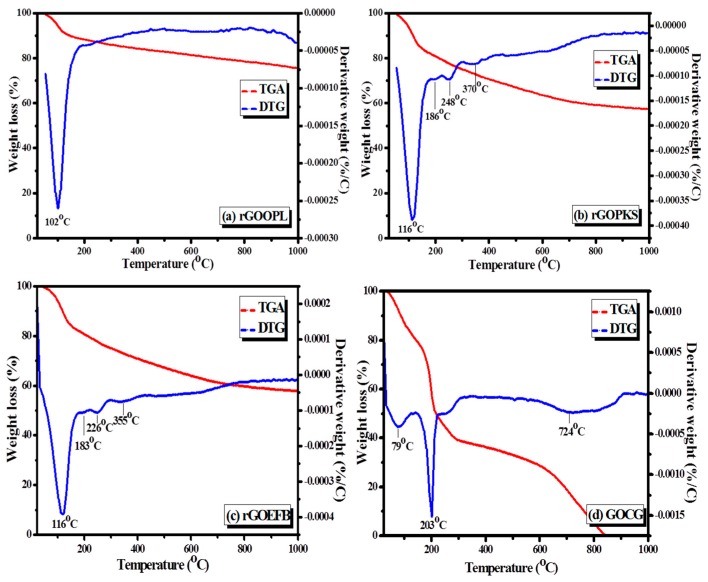
TGA/DTG thermograms of the (**a**) rGOOPL, (**b**) rGOPKS, (**c**) rGOEFB and (**d**) GOCG samples.

**Figure 11:**
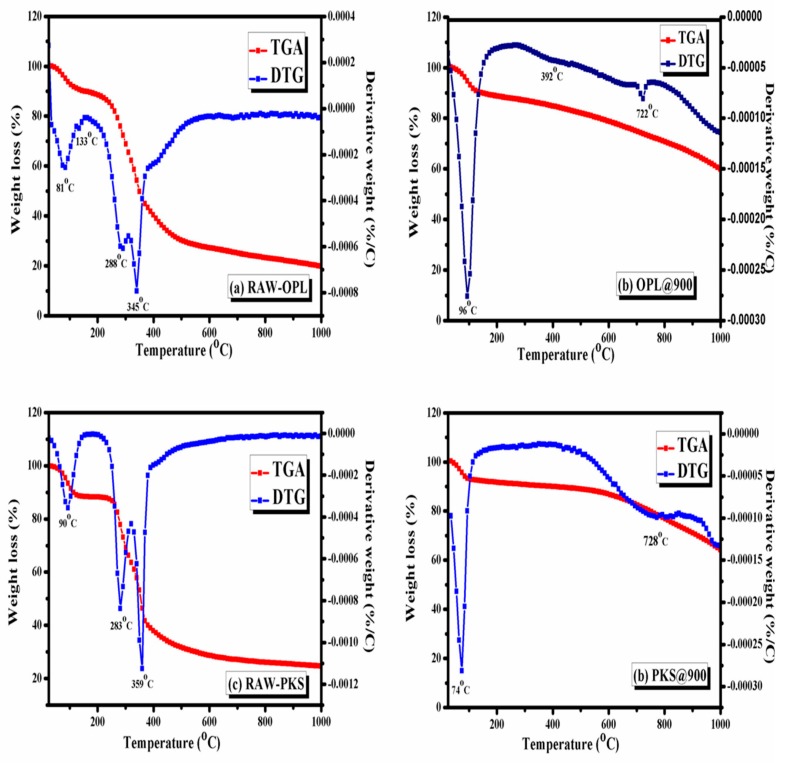
TGA/DTG thermograms of (**a**) the raw OPL sample; (**b**) the OPL sample carbonized at 900 °C; (**c**) the raw PKS sample; and (**d**) the as-carbonized PKS samples at 900 °C.

Thermogravimetric analysis (TGA/DTG) of the materials is presented in Figure 10 and Figure 11. The analysis was carried out to test the thermal stabilities of GO and rGO platelets. The result showed major weight losses between 102 and 370 °C. This is attributed to H_2_O (steam), CO and CO_2_ gas release from the mainly kinetically unstable functional groups. Concurrently, a moderate mass loss was also noticed between 375 and 900 °C and may be due to the elimination of more stable functionalities. By comparison, rGOOPL had the least weight loss; rGOPKS and rGOEFB had nearly similar weight losses; and GOCG had the highest among them.

It can be recalled that the starting materials (precursors) used in this study were typical plant or biomass materials that were generally known to contain significant amount of lignin, hemicelluloses and cellulose in their structures. Hence, decomposition of these materials as they are heated will give a good understanding of the thermal behavior of the as-generated materials. During the thermal decay process, at the first stage (below 200 °C), a slight weight loss of less than 10% of the biomass materials was observed, due to moisture loss (dehydration) and thermal decomposition reactions to form volatiles and organic molecules. It was reported that thermal decomposition of hemicellulose occurs at temperatures ranging from 150 to 350 °C [60]. In the same report, it was also expressed that cellulose decomposes at temperatures ranging between 275 and 350 °C, while lignin decomposes at 250 and 500 °C, which usually occurs at much higher temperatures than the others due to its heavy cross-linked structure. Therefore, as the decomposition temperature rose above 280 °C, the peaks observed at around 288, 345 and 359 °C (Figure 11) may possibly be due to volatiles resulting from the decomposition of inorganic materials, hemicellulose and cellulose, respectively. Based on the results, it was inferred that PKS was the most difficult to breakdown during the thermal decay process due to its high content of lignin as compared to OPL and EFB.

### 3.6. BET and N_2_ Adsorption/Desorption Isotherm Analyses

**Figure 12:**
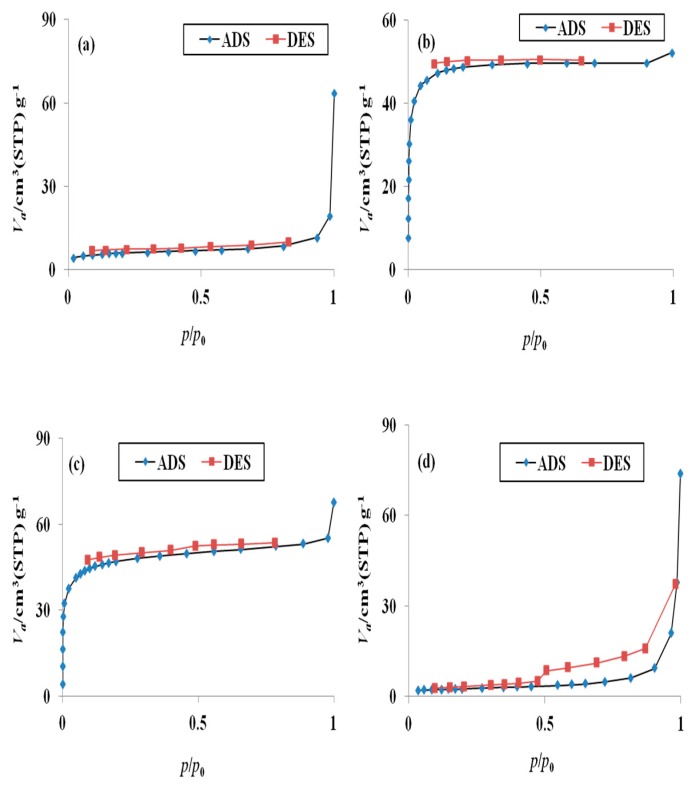
Nitrogen adsorption-desorption isotherms of (**a**) rGOOPL, (**b**) rGOPKS, (**c**) rGOEFB and (**d**) GOCG samples.

The surface area and pore volume of the samples were characterized by the BET-N_2_ adsorption/desorption isotherms analyses (Table 2 and Figure 12). The lower results observed for the rGOOPL and rGOCG samples could be attributed to the incomplete restacking of graphene-like material layers and nearby blocked pores. It is obvious that rGO is prone to re-stacking owing to active π-π interactions and van der Waals forces connecting the planar basal planes of GO sheets [61].

During the absorption process, gas is absorbed on the surface of the samples, and the applied pressure and volume of the gas adsorbed were recorded. Thus, N_2_ adsorption-desorption isotherms were obtained (Figure 12), and these were used to characterize the physical properties of the materials. The development of a porous structure contributed to the increase in the BET surface area of rGOPKS and rGOEFB, and this has been confirmed by N_2_ adsorption-desorption analysis. The open porous structure had possibly eased the diffusion of gaseous species produced by the decomposition of labile functional groups in rGOs during the low-temperature annealing reduction process of the GO [62]. In the same way, Figure 12a shows the characteristic of the Type III isotherm, which portrayed the typical feature of non-porous material. A similar feature is also observed for GOCG samples (Figure 12d). Concurrently, Figure 12b,c shows the feature of the Type I isotherm, which is typical for microporous type of carbon, consistent with the expectation of the nanoporous character of carbon material.

## 4. Conclusions

In our quest to find scalable and cost-efficient synthetic pathways to produce carbon nano-structured materials, particularly graphene-based materials, we have experimentally found that oil palm by-products such as palm kernel shells, oil palm leaves and empty fruit fibers, which are abundant bio-agricultural wastes in Malaysia, are very prominent, viable, environmentally-friendly and cost-efficient carbon sources for both GO and rGO preparation. The project comes at a time when the world is moving towards ‘green technology’. Therefore, the research could be used to stimulate economic growth in Malaysia while mitigating environmental degradation. This perfectly matched with the popular “From Waste to Wealth” mantra, which is aimed towards zero-waste industry. Therefore, the as-prepared materials could be used in various advanced technological applications based on the excellent properties associated with them. According to the experimental graphitization studies carried out in this work, we found that Raman spectroscopy and XRD techniques are excellent investigative tools to study the type and internal structure of graphitic materials produced from oil palm wastes.

Subsequently, taking into consideration the I_G_/I_D_ ratio of the resulting materials, which trail in the order of rGOOPL < rGOPKS < rGOCG < rGOEFB with the I_G_/I_D_ values of 1.06, 1.14, 1.16 and 1.20, respectively, we thus understand that an increase in the I_G_/I_D_ results in the increasing of the degree of the graphitization of the materials and high surface area (which also followed the same trend in the majority of the samples). Rationally, the acids (H_2_SO_4_; H_3_PO_4_; H_2_O_2_ and KMnO_4_) used as the chemical oxidation reagents of the starting materials and combined with the low-temperature annealing reduction of the graphene oxide had played a key role in the surface area and carbon deposition efficiency of the resulting materials.

We also noted initially that as the temperature increases during the analytical pyrolysis or carbonization of the starting materials prior to GOs and rGOs synthesis, the G-band became more prominent in the Raman spectra. The results also showed that the carbon yield rate decreased as the carbonization temperatures increased. More so, the 002 planes at about 2θ = 23. 6° from the XRD results also give evidence for the graphitic nature of the rGO samples.

## Figures and Tables

**Table 1:** I_D_, I_G_ and I_G_/I_D_ values of the as-prepared graphene oxide and reduced graphene oxide samples calculated from the Raman analysis.

S/N	rGO and GO Samples	I_D_	I_G_	I_G_/I_D_
1	rGOOPL	0.92	0.98	1.06
2	rGOPKS	0.87	0.99	1.14
3	rGOEFB	0.80	0.96	1.20
4	GOCG	0.83	0.97	1.16

**Table 2:** Surface area and pore volumes of graphene oxide and reduced graphene oxides prepared using various oil palm waste precursors.

rGO and GO Samples	BET Surface Area (m^2^/g)	Average Pore Diameter (nm)	Total Pore Volume (cm^3^/g)
rGOOPL	15	14	0.06
rGOPKS	114	3	0.08
rGOEFB	117	3	0.10
GOCG	8	41	0.08

## References

[1] Abdullah N., Sulaiman F., Matovic M.D. (2013). The oil palm wastes in Malaysia. Biomass Now—Sustainable Growth and Use.

[2] Umar M.S., Jennings P., Urmee T. (2014). Generating renewable energy from oil palm biomass in Malaysia: The Feed-in Tariff policy framework. Biomass Bioenergy.

[3] Shuit S.H., Tan K.T., Lee K.T., Kamaruddin A.H. (2009). Oil palm biomass as a sustainable energy source: A Malaysian case study. Energy.

[4] Deng J., You Y., Sahajwalla V., Joshi R.K. (2016). Transforming waste into carbon-based nanomaterials. Carbon.

[5] Deng J., Li M., Wang Y. (2016). Biomass-derived carbon: Synthesis and application on energy storage and conversion. Green Chem..

[6] Bonaccorso F., Colombo L., Yu G., Stoller M., Tozzini V., Ferrari A.C., Ruoff R.S., Pellegrini V. (2015). 2D materials. Graphene, related two-dimensional crystals, and hybrid systems for energy conversion and storage. Science.

[7] Gilje S., Han S., Wang M., Wang K.L., Kaner R.B. (2007). A chemical route to graphene for device applications. Nano Lett..

[8] Zhu Y., Murali S., Stoller M.D., Ganesh K.J., Cai W., Ferreira P.J., Pirkle A., Wallace R.M., Cychosz K.A., Thommes M. (2011). Carbon-based supercapacitors produced by activation of graphene. Science.

[9] Wang X., Shi Y. (2014). Fabrication techniques of graphene nanostructures. Nanofabr. Appl. Renew. Energy.

[10] Ferrari A.C., Meyer J.C., Scardaci V., Casiraghi C., Lazzeri M., Mauri F., Piscanec S., Jiang D., Novoselov K.S., Roth S. (2006). Raman spectrum of graphene and graphene layers. Phys. Rev. Lett..

[11] Xing T., Sunarso J., Yang W., Yin Y., Glushenkov A.M., Li L.H., Howlett P.C., Chen Y. (2013). Ball milling: A green mechanochemical approach for synthesis of nitrogen doped carbon nanoparticles. Nanoscale.

[12] Li Z.Q., Lu C.J., Xia Z.P., Zhou Y., Luo Z. (2007). X-ray diffraction patterns of graphite and turbostratic carbon. Carbon.

[13] Bae S., Kim H., Lee Y., Xu X., Park J.-S., Zheng Y., Balakrishnan J., Lei T., Ri Kim H., Song Y. (2010). Roll-to-roll production of 30-inch graphene films for transparent electrodes. Nat. Nanotechnol..

[14] Geim A.K., Novoselov K.S. (2007). The Rise of Graphene. Nat. Mater..

[15] Sur U.K. (2012). Graphene: A rising star on the horizon of materials science. Int. J. Electrochem..

[16] Sun M.J., Cao X., Cao Z. (2016). Si(C~C)4-based single-crystalline semiconductor: Diamond-like superlight and superflexible wide-bandgap material for the UV photoconductive device. ACS Appl. Mater. Interfaces.

[17] Jacob M.V., Rawat R.S., Ouyang B., Bazaka K., Kumar D.S., Taguchi D., Iwamoto M., Neupane R., Varghese O.K. (2015). Catalyst-free plasma enhanced growth of graphene from sustainable sources. Nano Lett..

[18] Ruan G., Sun Z., Peng Z., Tour J.M. (2011). Growth of graphene from food, insects, and waste. ACS Nano.

[19] Kretinin A.V., Cao Y., Tu J.-S., Yu G., Jalil R., Novoselov K.S., Haigh S., Gholinia A., Mishchenko A., Lozada M. (2014). Electronic properties of graphene encapsulated with different 2D atomic crystals. Nano Lett..

[20] Yuan B., Shi Y., Mu X., Wang J., Xing W., Liew K.M., Hu Y. (2016). A facile method to prepare reduced graphene oxide with a large pore volume. Mater. Lett..

[21] Brodie B.C., Trans P., Lond R.S. (1859). On the atomic weight of graphite. Philos. Trans. R. Soc. Lond..

[22] Hummers W.S., Offeman R.E. (1958). Preparation of graphitic oxide. J. Am. Chem. Soc..

[23] Jiang Y., Shao H., Li C., Xu T., Zhao Y., Shi G., Jiang L., Qu L. (2016). Versatile graphene oxide putty-like material. Adv. Mater..

[24] Liao L., Lin Y., Bao M., Cheng R., Bai J., Liu Y., Qu Y., Wang K.L. (2010). High-speed graphene transistors with a self-aligned nanowire gate. Nature.

[25] Berger C., Mayou D., Li T., Hass J., Marchenkov A.N., Conrad E.H., First P.N., de Heer W.A. (2006). Electronic confinement and coherence in patterned epitaxial graphene. Science.

[26] Dimiev A., Eigler S., Dimiev A.M., Eigler S. (2017). Graphene Oxide: Fundamentals and Applications.

[27] Tian Z., Yu P., Lowe S.E., Pandolfo A.G., Gengenbach T.R., Nairn K.M., Song J., Wang X., Zhong Y.L., Li D. (2017). Facile electrochemical approach for the production of graphite oxide with tunable chemistry. Carbon.

[28] Yu P., Tian Z., Lowe S.E., Song J., Ma Z., Wang X., Han Z.J., Bao Q., Simon G.P., Li D. (2016). Mechanically-assisted electrochemical production of graphene oxide. Chem. Mater..

[29] Yu C., Wu Y., Liu X., Yao B., Fu F., Gong Y., Rao Y., Chen Y. (2016). Graphene oxide deposited microfiber knot resonator for gas sensing. Opt. Mater. Express.

[30] Han J.W., Kim E., Kim J., Han J.W., Kim J. (2015). Reduction of graphene oxide by resveratrol: A novel and simple biological method for the synthesis of an effective anticancer nanotherapeutic molecule. Int. J. Nanomed..

[31] Morimoto N., Kubo T., Nishina Y. (2016). Tailoring the oxygen content of graphite and reduced graphene oxide for specific applications. Sci. Rep..

[32] Salimian M., Ivanov M., Deepak L., Petrovykh D.Y., Bdikin I., Ferro M., Kholkin A., Goncalves G. (2015). Synthesis and characterization of reduced graphene oxide/spiky nickel nanocomposite for nanoelectronic applications. J. Mater. Chem. C.

[33] Usman M.S., Hussein M.Z., Fakurazi S., Ahmad Saad F.F. (2017). Gadolinium-based layered double hydroxide and graphene oxide nano-carriers for magnetic resonance imaging and drug delivery. Chem. Cent. J..

[34] Thangavelu R.R. (2013). Synthesis and characterization of reduced graphene oxide. Adv. Mater. Res..

[35] Dreyer D.R., Park S., Bielawski W., Ruoff R.S. (2010). The chemistry of graphene oxide. Chem. Soc. Rev..

[36] Pei S., Cheng H. (2012). The reduction of graphene oxide. Carbon.

[37] Feng H., Cheng R., Zhao X., Duan X., Li J. (2013). Reduced graphene oxide. Nat. Commun..

[38] Stankovich S., Dikin D.A., Piner R.D., Kohlhaas K.A., Kleinhammes A., Jia Y., Wu Y., Nguyen S.T., Ruoff R.S. (2007). Synthesis of graphene-based nanosheets via chemical reduction of exfoliated graphite oxide. Carbon.

[39] Shin B.H., Kim K.K., Benayad A., Yoon S., Park K., Jung I., Jin M.H., Jeong H., Kim J.M., Choi J. (2009). Efficient reduction of graphite oxide by sodium borohydride and its effect on electrical conductance. Adv. Funct. Mater..

[40] Bo Z., Shuai X., Mao S., Yang H., Qian J., Chen J., Yan J., Cen K. (2014). Green preparation of reduced graphene oxide for sensing and energy storage applications. Sci. Rep..

[41] Fernández-Merino M.J., Guardia L., Paredes J.I., Solís-Fernández P., Martínez-Alonso A., Tasco J.M.D. (2010). Vitamin C is an ideal substitute for hydrazine in the reduction of graphene oxide suspensions. J. Phys. Chem. C.

[42] Yang D., Velamakanni A., Bozoklu G., Park S., Stoller M., Piner R.D., Stankovich S., Jung I., Field D.A., Ventrice C.A. (2009). Chemical analysis of graphene oxide films after heat and chemical treatments by X-Ray photoelectron and micro-raman spectroscopy. Carbon.

[43] Marcano D.C., Kosynkin D.V., Berlin J.M., Sinitskii A., Sun Z., Slesarev A., Alemany L.B., Lu W., Tour J.M. (2010). Improved synthesis of graphene oxide. ACS Nano.

[44] Akhavan O., Bijanzad K., Mirsepah A. (2014). Synthesis of graphene from natural and industrial carbonaceous wastes. RSC Adv..

[45] Adolfsson K.H., Hassanzadeh S., Hakkarainen M. (2015). Valorization of cellulose and waste paper to graphene oxide quantum dots. RSC Adv..

[46] Sun Z., Yan Z., Yao J., Beitler E., Zhu Y., Tour J.M. (2010). Growth of graphene from solid carbon sources. Nature.

[47] Somanathan T., Prasad K., Ostrikov K., Saravanan A., Krishna V. (2015). Graphene oxide synthesis from agro waste. Nanomaterials.

[48] Peng W., Liu S., Sun H., Yao Y., Zhi L., Wang S. (2013). Synthesis of porous reduced graphene oxide as metal-free carbon for adsorption and catalytic oxidation of organics in water. J. Mater. Chem. A.

[49] Nasar M., Salisu N. (2014). Investigation of effect of KBr matrix on drift infrared spectra of some minerals. ChemSearch J..

[50] Ferrari A.C. (2007). Raman spectroscopy of graphene and graphite: Disorder, electron—Phonon coupling, doping and nonadiabatic effects. Solid State Commun..

[51] Tuinstra F., Koenig L. (1970). Raman spectrum of graphite. J. Chem. Phys..

[52] Ferrari A., Robertson J. (2000). Interpretation of raman spectra of disordered and amorphous carbon. Phys. Rev. B.

[53] Cho N.H., Veirs D.K., Ager J.W., Rubin M.D., Hopper C.B., Cho N.H., Veirs D.K., Iii J.W.A., Rubin M.D., Hopper C.B. (1992). Effects of substrate temperature on chemical structure of amorphous carbon films carbon of substrate films temperature on chemical structure of amorphous. J. Appl. Phys..

[54] Tay B.K., Shi X., Liu E.J., Tan H.S., Cheah L.K. (1999). Effects of substrate temperature on the properties of tetrahedral amorphous carbon films. Thin Solid Films.

[55] Budde H., Coca Lopez N., Shi X., Ciesielski R., Lombardo A., Yoon D., Ferrari A.C., Hartschuh A. (2016). Raman radiation patterns of graphene. ACS Nano.

[56] De Silva K.S.B., Gambhir S., Wang X.L., Xu X., Li W.X., Officer D.L., Wexler D., Wallace G.G., Dou S.X. (2012). The effect of reduced graphene oxide addition on the superconductivity of MgB2. J. Mater. Chem..

[57] Gupta B., Kumar N., Panda K., Kanan V., Joshi S., Visoly-Fisher I. (2017). Role of oxygen functional groups in reduced graphene oxide for lubrication. Sci. Rep..

[58] Dimiev A.M., Alemany L.B., Tour J.M. (2013). Graphene oxide. Origin of acidity, its instability in water, and a new dynamic structural model. ACS Nano.

[59] Kim H., Park K.-Y., Hong J., Kang K. (2014). All-graphene-battery: Bridging the gap between supercapacitors and lithium ion batteries. Sci. Rep..

[60] Chen W., Kuo P. (2010). A study on torrefaction of various biomass materials and its impact on lignocellulosic structure simulated by a thermogravimetry. Energy.

[61] Kim K.H., Yang M., Cho K.M., Jun Y.-S., Lee S.B., Jung H.-T. (2013). High quality reduced graphene oxide through repairing with multi-layered graphene ball nanostructures. Sci. Rep..

[62] Lin Z., Waller G.H., Liu Y., Liu M., Wong C.P. (2013). 3D Nitrogen-doped graphene prepared by pyrolysis of graphene oxide with polypyrrole for electrocatalysis of oxygen reduction reaction. Nano Energy.

